# Spatial transmission network construction of influenza-like illness using dynamic Bayesian network and vector-autoregressive moving average model

**DOI:** 10.1186/s12879-021-05769-6

**Published:** 2021-02-10

**Authors:** Jianqing Qiu, Huimin Wang, Lin Hu, Changhong Yang, Tao Zhang

**Affiliations:** 1 Department of Epidemiology and Health Statistics, West China School of Public Health and West China Fourth Hospital, Sichuan University, Chengdu, Sichuan China; 2 Sichuan Center for Disease Control and Prevention, Chengdu, China

**Keywords:** Influenza, Spatial transmission network, Dynamic Bayesian network, Vector autoregressive moving average model, Spatio-temporal route

## Abstract

**Background:**

Although vaccination is one of the main countermeasures against influenza epidemic, it is highly essential to make informed prevention decisions to guarantee that limited vaccination resources are allocated to the places where they are most needed. Hence, one of the fundamental steps for decision making in influenza prevention is to characterize its spatio-temporal trend, especially on the key problem about how influenza transmits among adjacent places and how much impact the influenza of one place could have on its neighbors. To solve this problem while avoiding too much additional time-consuming work on data collection, this study proposed a new concept of *spatio-temporal route* as well as its estimation methods to construct the influenza transmission network.

**Methods:**

The influenza-like illness (ILI) data of Sichuan province in 21 cities was collected from 2010 to 2016. A joint pattern based on the dynamic Bayesian network (DBN) model and the vector autoregressive moving average (VARMA) model was utilized to estimate the spatio-temporal routes, which were applied to the two stages of learning process respectively, namely structure learning and parameter learning. In structure learning, the first-order conditional dependencies approximation algorithm was used to generate the DBN, which could visualize the spatio-temporal routes of influenza among adjacent cities and infer which cities have impacts on others in influenza transmission. In parameter learning, the VARMA model was adopted to estimate the strength of these impacts. Finally, all the estimated spatio-temporal routes were put together to form the final influenza transmission network.

**Results:**

The results showed that the period of influenza transmission cycle was longer in Western Sichuan and Chengdu Plain than that in Northeastern Sichuan, and there would be potential spatio-temporal routes of influenza from bordering provinces or municipalities into Sichuan province. Furthermore, this study also pointed out several estimated spatio-temporal routes with relatively high strength of associations, which could serve as clues of hot spot areas detection for influenza surveillance.

**Conclusions:**

This study proposed a new framework for exploring the potentially stable spatio-temporal routes between different places and measuring specific the sizes of transmission effects. It could help making timely and reliable prediction of the spatio-temporal trend of infectious diseases, and further determining the possible key areas of the next epidemic by considering their neighbors’ incidence and the transmission relationships.

**Supplementary Information:**

The online version contains supplementary material available at 10.1186/s12879-021-05769-6.

## Background

Influenza is an acute respiratory infection caused by influenza viruses [1]. According to the globally estimated disease burden attributed to the influenza, it is estimated there are about 1 billion seasonal influenza cases per year on average, among which three million cases are serious, resulting in 250,000 to 500,000 deaths [2].

Although vaccination is one of the main countermeasures against influenza epidemic [3, 4], some drawbacks still exist. For example, various influenza virus strains are prone to undergo antigen drift and antigen conversion, which often makes vaccine-induced immunity wane over the course of a season. Besides, there exists a natural process of decreasing trend of antibody titers after vaccination [5], suggesting people need to be vaccinated periodically, leading to a huge burden to low-and-middle income countries. Therefore, to compensate for the huge cost by prevention strategies, it is quite necessary to make informed prevention decisions to guarantee that limited resources are allocated to the places where they are most needed [6]. One of the fundamental steps for decision making in influenza prevention is to characterize its spatio-temporal trend, especially on the key problem about how influenza transmits among adjacent places and how much impact the influenza of one place could have on its neighbors. To solve such problems, Fu et al. [7] developed complex networks with population contact data to predict the epidemic trend in a mathematical way. Recently, Pei et al. [8] utilized accessible human mobility data and a metapopulation model for predicting the spatial transmission of influenza in the United States. In addition, another study has utilized birds migration network to predict the trajectory of avian influenza [9]. Those methods require personal contact data including person mobility data, traffic data, avian mobility data and so on. However, there will be some obstacles on the availability of those data, and collecting those data will be too time-consuming to make rapid strategies of influenza prevention.

To overcome these obstacles of previous researches, this study proposed a new concept called *spatio-temporal route* to display the potential transmission directions of influenza and to measure the sizes of those transmission effects. This concept was defined as the time-lagged association among influenza surveillance data across different places. In addition, since the estimated *spatio-temporal route* only depends on surveillance data and does not necessarily need personal contact data, it will be convenient to be used for exploring the transmission network with real-world surveillance data within statistical framework. For illustration purpose, this study selected Sichuan province in China as an example, but the concept of *spatio-temporal route* as well as its estimation methods mentioned below could also be applied to other places.

## Materials and methods

### Data preparation

The data of this study came from all the sentinels of influenza-like illness (ILI) of Sichuan province from 2010 to 2016. Sentinel surveillance is one of the important measures for infectious diseases surveillance. According to the unified deployment of the National Center for Disease Control and Prevention (CDC) and the real-world situation of Sichuan province, the sentinel surveillance of influenza-like illness is simultaneously conducted in each of the 21 cities in Sichuan province by hospitals, CDCs, and primary health service institutions. According to the report requirement, the medical staffs of monitoring clinics in sentinels recorded the number of ILI and the total number of outpatients in each age group in each department every day, and uploaded the data to the *China Influenza Surveillance Information System* before midnight every Monday. For this study, the definition of influenza-like illness (ILI) referring to WHO [10] was as follows: a case measured fever of ≥38 C° and cough; with onset within the last 10 days. Besides, to estimate the absolute ILI case number in city *j*, we collected the data of yearly number of medical outpatients in each city of Sichuan from S*ichuan Health Statistics Yearbook 2010 ~ 2012* and *Sichuan Health and Family Planning Statistical Yearbook 2013 ~ 2016*. We defined the ILI case number in city *i* and week *t* as *ILI*(*i*, *t*) so that for any city *i* (i.e., when *i* is fixed), *ILI*(*i*, *t*) (*t* = 1, 2, 3, …) could be regarded as time series, and the task of this study was to model the time-lagged correlation between any of the two time series *ILI*(*i*, *t*) and *ILI*(*j*, *t*) (*i* ≠ *j*, *t* = 1, 2, 3,…).

### The estimation of spatio-temporal routes

#### The definition of spatio-temporal routes

Some previous researches engaged in constructing the influenza transmission network by temporal and spatial statistics [11–16]. For example, Alonso WJ [16] used Fourier decomposition to find a seasonal southward traveling wave of influenza across Brazil originating from equatorial and low population regions in March–April and moving towards temperate and highly popular regions over a 3-month period. In addition, Paul and Held [17] proposed a random effect model (the *epidemic-endemic* model) to consider the transmission effects of neighboring places. From the statistical point of view, the phenomenon that influenza transmitting from place A to place B could be reflected by the time-lagged association. Equivalently, the time-lagged association could also be visualized by A → B, where the directed arc indicated that node A (i.e., influenza in place A) had a time-lagged effect on node B (i.e., influenza in place B), and we defined such directed arc as *spatio-temporal route*. Furthermore, if the time-lagged associations existed in more than two places in the overall study area, then all the arcs would interweave into a network. Such a network could show how historical influenza in one place would influence its neighbors in the near future so as to make some possible inferences on the temporal and spatial transmission features of influenza. To this end, we defined this network as *spatial transmission network* because it was essentially a set of *spatio-temporal routes*.

More precisely, the spatio-temporal routes could also be defined in a mathematical way. Let ***X*** = {*ILI* (*i*,*r*)}be the set of all the influenza data from different places. Define ***A*** the set of arcs between any two places in set ***X*** and then the spatio-temporal routes could be defined as network ***G*** = (***X***, ***A***). Specifically, the network ***G*** contains two types of information. The first type was the structure information, which was related to arc existence as well as its direction for any pair of two nodes in ***G***. The second type was the parameter information, which measured the strengths of associations among different nodes. Correspondingly, the estimation of spatio-temporal routes consisted of structure learning and parameter learning, which was dedicated to drawing the structure and parameter information respectively from the original data. Specifically, this study used the dynamic Bayesian network (DBN) model for structure learning and the vector autoregressive moving average (VARMA) model for parameter learning. More details were given as below.

#### The structure learning of spatio-temporal routes by the DBN

The DBN is a dynamic directed acyclic graph (DAG) using nodes and arcs to express the conditional probabilistic dependencies between a set of time series [18]. In the DBN model, an arc is drawn between two variables at successive time points. For example, from *ILI*(*i*, *t-*1) to *ILI*(*j*, *t*), which means the ILI cases of city *j* at time *t* (e.g., the current week) are conditionally dependent on the ILI cases of city *i* at time (*t*-1) (e.g., 1 week ago) given the remaining variables at the past time points. Due to its good theoretical properties, the DBN model was used to characterize the gene regulatory network by characterizing the time-lag associations of multiple gene expression data [19, 20]. Recently, our previous work had also used simulation studies to prove that the DBN could be very well applied to the infectious disease surveillance data even when confronted with some rigorous challenges such as high noise, nonlinear correlation, small sample and latent variables [21]. Therefore, this study used the DBN model for the structure learning of influenza spatio-temporal routes.

In particular, we estimated the DBN model by using the first-order conditional dependencies approximation algorithm [22]. It implemented DBN learning as a two-step procedure. At the first step, it learned a DAG encoding first-order partial dependence relationships. Then it inferred the real network structure of the DBN using the graph from the first step [18]. Once the structure learning of influenza spatio-temporal routes was completed, it could be used to infer which cities have impact on others in influenza transmission. Then the next step was to further estimate the strength of these impacts by the means of parameter learning.

#### The parameter learning of spatio-temporal routes by the VARMA model

As mentioned above, the parameter learning of influenza spatio-temporal routes was required to quantify how the current ILI cases in one place were impacted by the past ILI cases in other places. To this end, the multivariate time series (MTS) models were used for parameter learning [23]. Furthermore, it has been proven that the DBN is mathematically equivalent to the vector-autoregressive model (VAR) [24] (i.e., one of the most commonly used MTS model), which again shed light upon the application of the VAR model to parameter learning of influenza spatio-temporal routes. Specifically, the basic formula of VAR(*p*) was:

$$ {\boldsymbol{ILI}}_t=\sum \limits_{i=1}^p{\boldsymbol{A}}_i{\boldsymbol{ILI}}_{t-i}+{\boldsymbol{\varepsilon}}_t, $$

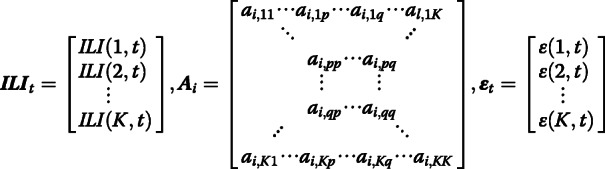

where *K* stood for the overall number of the involved places (i.e., *K* = 21 in this study since there were 21 cities in Sichuan province), *ILI*(*i*, *t*) was defined in Section 2.1 and *ε*(*i*, *t*) was defined as the residual of the fitted model for ILI cases in city *i* at time *t*. For the VAR model, it assumed that at any given time *t*, all the residuals***ε*** (*1, t*), *ε* (2, *t*), …, *ε*(*K*, *t*) were independent with each other. However, in the real-world situation of influenza transmission, since some important information of influenza transmission factors (e.g., population density, the effectiveness of influenza transmission in the city, festival effects and so on) may not be captured, consequently the assumption of residual independence would be violated. Therefore, this study improved the VAR model to the VARMA model as below [25]:

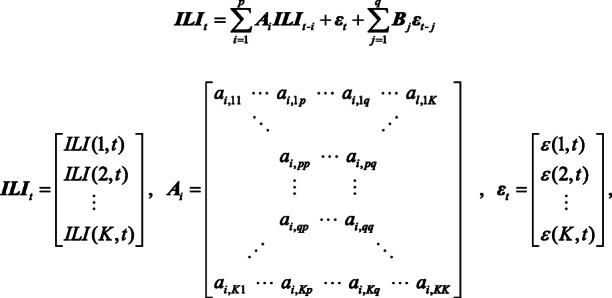

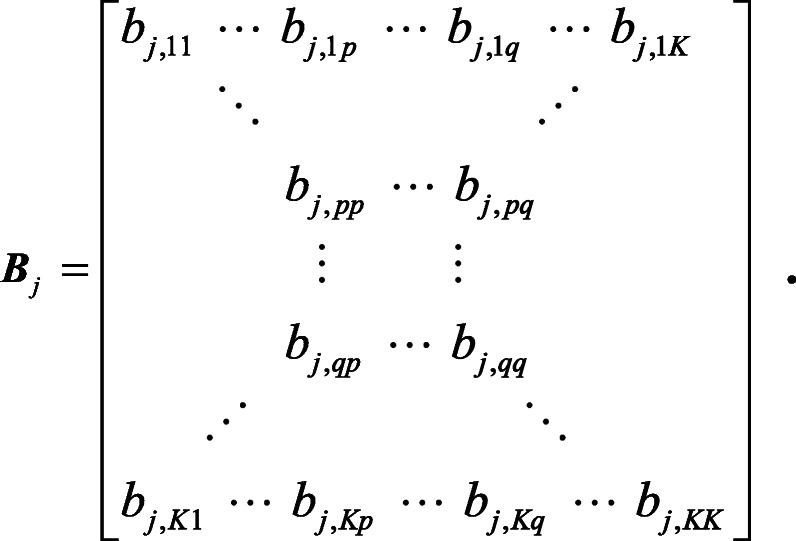





Compared with the VAR model, the VARMA model could compensate for the violation of residual independence assumption by adding the moving average term $\sum \limits_{j=1}^q{\boldsymbol{B}}_j{\boldsymbol{\varepsilon}}_{t-j}$ to extract the remaining dependency information between residuals. Therefore, it was plausible that the VARMA model could complete the parameter learning while reducing the error of parameter estimation as possible.

During the estimation of spatio-temporal routes, both the DBN and the VARMA models could be implemented in R 3.6.3, a free sofware environment for statistical computing and graphics. The DBN model was implemented using the {G1DBN} package and the VARMA model was built by the {MTS} package. All the packages were downloaded from the Comprehensive R Archive Network (CRAN) at http://cran.r-project.org/ and installed in advance. Additionally, the time series plots of ILI% in Sichuan and the maps of Sichuan were generated by us with R software.

## Results

### The ILI% in Sichuan between 2010 and 2016

**Fig. 1:**
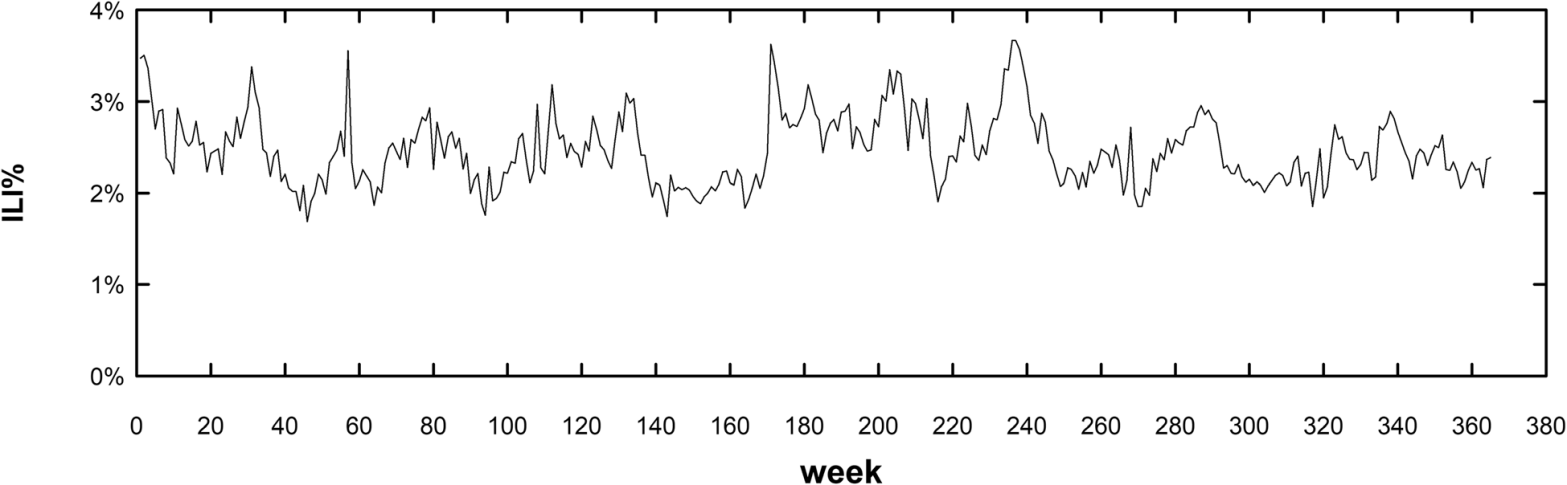
The ILI% time series in Sichuan surveillance sentinels, 2010–2016. *The time series of ILI% in Sichuan was generally stable from 2010 to 2016, with maximum of 3.66% at week 237 and minimum of 1.69% at week 46

**Fig. 2:**
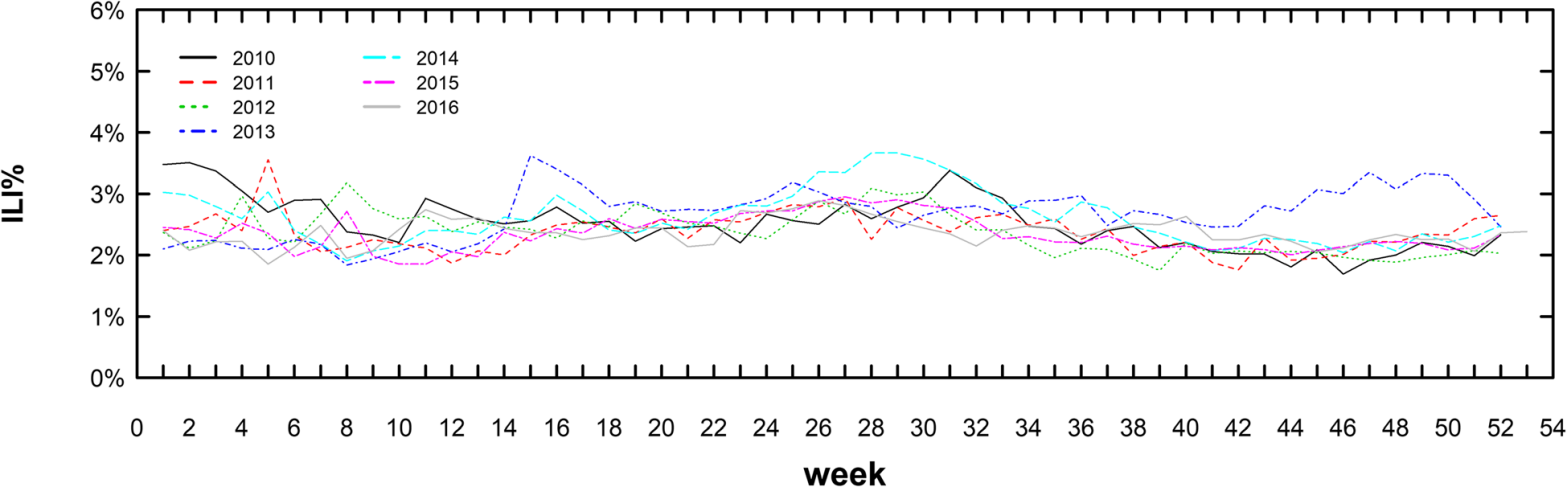
The yearly ILI% time series in Sichuan surveillance sentinels, 2010–2016. *The time series of ILI% in different years were almost similar

The total number of outpatients in Sichuan surveillance sentinels from 2010 to 2016 was 31,898,487, and the total number of influenza-like cases in Sichuan was 784,984. As a result, the ILI% of Sichuan was 2.46% within 6 years. From 2010 to 2016, the cumulative ILI% was 2.51, 2.36, 2.38, 2.69, 2.60, 2.34 and 2.36%, respectively. The year with lowest ILI number was 2010 (84,766 visits), and highest was 2016 (137,945 visits); the lowest ILI% was in 2016 (2.36%) and the highest was in 2013 (2.69%). The 2010–2016 weekly ILI% distribution was shown in Fig. 1 and Fig. 2 below:

From Fig. 1 and Fig. 2, the weekly ILI% in Sichuan province from 2010 to 2016 was between 1.69 and 3.66%, which was consistent with the past years. From Fig. 2, it could be seen there was a slight peak in winter and spring (From the 47th to 52nd week, and from 1st to 9th week), similar to the result of the whole country [26].

### Results of structure learning of possible influenza spatio-temporal routes

**Fig. 3:**
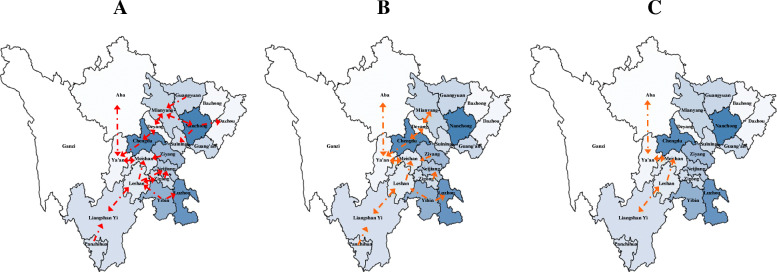
The Sequential-week lagged spatio-temporal routes of influenza among different cities. **a** 1-week lagged **b** 2-week lagged **c** 3-week lagged. *The No. of influenza spatio-temporal routes showed a decreasing trend in 3-week period. The maps of Sichuan were generated by R software

Combining the longest incubation period of influenza (i.e. 2 weeks [27]) and our exploratory work, we set 1-week lag, 2-week lag and 3-week lag as lags of time order respectively, and the final influenza spatial transmission network was obtained as Fig. 3:

In the perspective of prevention for influenza, from Fig. 3, it could be observed that during 3-week influenza transmission cycle, the number of suspected influenza spatio-temporal routes in Sichuan province showed a decreasing trend. Hence, it could be speculated that the period of influenza transmission cycle was longer in Western Sichuan and Chengdu Plain and shorter in Northeastern Sichuan, suggesting that we should consider extending the period for influenza prevention and control in Western Sichuan and Chengdu Plain appropriately compared with Northeastern Sichuan.

To verify the robustness of structure learning across different ages and years, we fitted DBN models within each data subset of different age groups (0–5 year age group, 5–15 year age group, 15–25 year age group, 25–60 year age group and 60+ year age group) and each year (2010, 2011, 2012, 2013, 2014, 2015 and 2016). In terms of age stratification analysis, we found that the structure learning results in each subset were almost consistent with the original result shown in Fig. 3, except for the result in 0–5 year age group and 60+ age year group. Such differences might be explained by the variance of population mobility across different age groups, because the aged and the infants were much less likely to go on a long journey than the young adults. As for subset analysis within each year, the results in 2010, 2011, 2012, 2013 and 2014 were also consistent with the structure in Fig. 3 A to some extent, but in 2015 and 2016 the results were a bit different. As the Fig. 2 implied, the ILI%s in those 2 years were lower, so it was plausible to infer that the lower sample size weakened the statistical power of structure learning in 2015 and 2016, resulting in inconsistencies in 2015 and 2016.

**Fig. 4:**
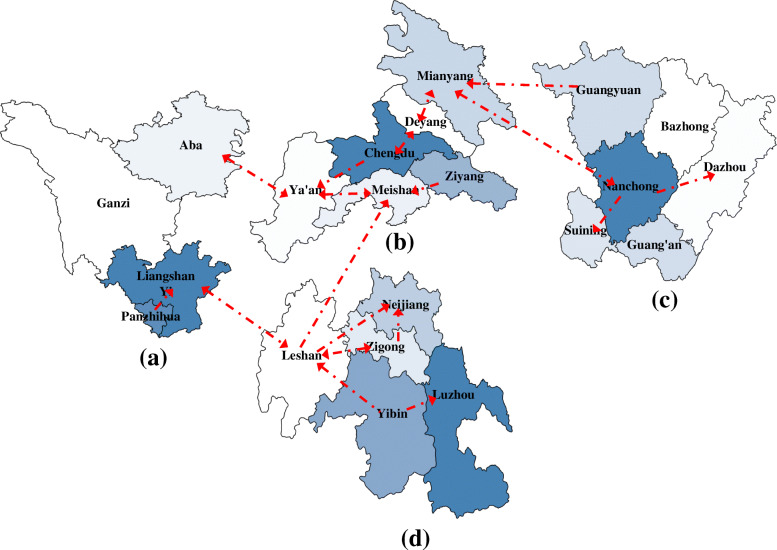
1-week lagged influenza transmission network in different subregions of Sichuan province. (**a**) Western Sichuan subregion (**b**) Chengdu Plain subregion (**c**) Northeastern Sichuan subregion (**d**) Southern Sichuan subregion. *There were four cities with single-direction arrows pointing to other cities in the province while with no city in the province pointing to themselves, i.e., Ziyang, Guangyuan, Yibin and Panzhihua. The map of Sichuan was generated by R software

More importantly, it could be seen from Fig. 4 that there were four cities with single-direction arrows pointing to other cities in the province while with no city in the province pointing to themselves, i.e., Ziyang, Guangyuan, Yibin and Panzhihua. In addition, all the four cities are bordering cities in Sichuan province and essential transportation hubs connecting to other adjacent provinces. Furthermore, influenza is a typical human-to-human infectious disease, so transportation could play an important role in the transmission of influenza logically, which has already been confirmed by a large number of studies. For example, Hidenori [28] found that traffic control could delay the spread of flu during peak flu periods in a simulation study. Therefore, it was plausible to speculate that there would be potential spatio-temporal routes of influenza from bordering provinces or municipalities into Sichuan province.

In addition, to verify the rationality of this speculation, it was interesting to show the high correlation between the number of estimated influenza spatio-temporal routes and the highway transportation capacities in each subregion of Sichuan province (Table 1). Except for the correlation in numbers, the relations between influenza and transportation could again be suggested by the coincidence of the estimated influenza spatio-temporal routes and the major highways in Sichuan. Taking Chengdu Plain subregion as an example, the Ziyang→Meishan influenza spatio-temporal routes coincided with the Suizimei Expressway; the Chengdu→Ya’an influenza spatio-temporal route was to some extent in accordance with the Chengya Expressway; the Guangyuan→Mianyang← → Deyang← → Chengdu→Ya’an influenza spatio-temporal route was possibly in line with the G5 Jingkun Expressway (the Mianguang section, the Chengmian section and the Chengya section); the Nanchong ← → Mianyang ← → Deyang ← → Chengdu influenza spatio-temporal route was expected to be brought by the Chengdemiannan Expressway. Besides, Leshan→Meishan← → Ya’an influenza spatio-temporal route was supposed to be related with the Leya Expressway, which was another important highway flowing through Ya’an. More examples about the coincidence of the estimated influenza spatio-temporal routes and the major highways in other subregions in Sichuan province could be seen in the Additional file 1. All these examples indicated that highway transportation might be a key factor underlying the estimated influenza spatio-temporal routes in Sichuan province.

**Table 1:** The number of estimated influenza spatio-temporal routes and the highway transportation capacities in each subregion of Sichuan province

Subregion	No. of the summaried estimated influenza spatio-temporal routes regardless of directions	No. of passengers by highway	No. of passenger turnovers (thousand person-kilometers)
**Western Sichuan**	3	184,650	7,868,210
**Northeastern Sichuan**	4	279,490	13,915,980
**Southern Sichuan**	7	342,710	15,535,290
**Chengdu Plain**	9	349,340	22,464,720

### Results of parameter learning of possible influenza spatio-temporal routes

The estimated parameters of influenza spatio-temporal routes were summarized in Table 2, and meanwhile the specific parameter learning results using the VARMA model were in Additional file 2. It could be seen that the median of the estimated parameters lied around zero, indicating that most of the influenza spatio-temporal routes were in general not obvious. Among the four bordering cities mentioned in Section 3.2, the spatio-temporal effect of Ziyang deserved special attention. In particular, the 1-week lagged spatio-temporal effect of Ziyang to Meishan was 0.35, and from Meishan to Ya’an was 0.07, implying that Ziyang played an essential role in terms of spatio-temporal effects. All these results showed that the estimated time-lagged relationships of influenza cases among these cities were relatively close, suggesting that the prevention of influenza transmission among those cities should be highlighted. In addition, it could be seen from Table 2 that from a lag of 1 week to a lag of 3 weeks, the degrees of dispersion were almost stable, implying the change of spatio-temporal effects might not be obvious.

**Table 2:** The estimated parameters of influenza spatio-temporal routes at sequential-lagged week

Lagged week	No. of possible spatio-temporal routes	Min	Lower Quartile	Median	Upper Quartile	Max	Interquartile Range
**1 week**	43	−0.8300	−0.1000	− 0.0100	0.0700	0.7500	0.1700
**2 week**	27	−0.4300	−0.0750	− 0.0300	0.1000	0.8400	0.1750
**3 week**	7	−0.9000	−0.0950	− 0.0100	0.0100	0.1100	0.1050
**Total**	77	−0.9000	−0.0925	− 0.0200	0.0700	0.8400	0.1625

## Discussion

This study proposed the concept of spatio-temporal route as well as its estimation methods to construct the influenza transmission network in a novel way. To our knowledge, this study may contribute to the infectious diseases surveillance in at least the following three ways.

1. This study initially proposed the concept of *spatio-temporal route* to better solve the problem of how to construct a potential spatial transmission network of influenza when mobility data is unavailable. On the one hand, in traditional epidemiology, there is a similar concept named the *route of transmission*, which mainly refers to the entire process experienced by pathogens in the external environment from the time they are discharged from infection sources to the time they invade new susceptible hosts [29]. Hence, one could judge by definition that it is impossible to answer the question of constructing a spatial transmission network by study on the route of transmission. On the other hand, in most of the latest researches about the influenza transmission network, the analytical models were based on theoretical physics or internet disciplines [30, 31], as well as the mobility information like the air transportation network. However, very few studies participated in constructing a spatial transmission network of influenza through the perspective of spatio-temporal distribution. Hence, the spatio-temporal routes not only help to clarify the parameters of interest in this study, but also provide a theoretical foundation for further researches to study the propagation and epidemic law of infectious diseases from the temporal and spatial dimensions.
2. This study also put forward a joint pattern based on DBN and VARMA models to estimate the spatio-temporal routes for the first time. Although neither of the two models was proposed by this study for the first time, this research has made full use of the theoretical properties and combined their advantages together. In particular, previous studies have proved the validity and robustness of DBN model when handling complicated data structure such as high dimension, high noise and nonlinearity [21], which served as a powerful guarantee to reveal the spatio-temporal correlations between the ILIs of different areas. In addition, the VARMA model has advantages in dealing with the potential confounders due to data unavailability in practice. Therefore, when the two models were combined, it was plausible that they would be well applied to infer influenza transmission network in the complicated real-world of influenza surveillance.
3. Another potential contribution of this study was to help making timely and reliable prediction of the spatio-temporal trend of influenza, and further determining the possible key areas for the next influenza epidemic outbreak. According to Stoto [32], a practical symptom surveillance system required *continuous surveillance data* (possibly multivariate data), *an alert generated by the application of predictive algorithms*, and *a prescribed process for how to respond to the alert*. To this end, it was promising that this study could help to improve the current influenza surveillance system in the following ways. Firstly, this study confirmed that our model could efficiently utilize continuous surveillance data of multiple places for influenza surveillance. Secondly, the results of this study revealed that some adjacent cities were indeed close to each other in ILI cases, which suggested that there might be some potential stable spatio-temporal routes among these cities so that the CDC could locate the key areas of the epidemics and send alarms when necessary. For example, as mentioned before, the spatio-temporal association between Ziyang to Meishan was relatively high. Therefore, if an influenza outbreak happened in Ziyang, one should pay attention to Meishan because our model reminded close relationship between them. Finally, on the basis of the previous early warning prevention and control policies, the local authorities receiving the alert could formulate specific prevention and control strategies according to the actual situation, such as identifying the key population for vaccination, enhancing inspection and quarantine as well as timely allocating health resources.

Although there were some interesting findings in this study, some limitations should also be acknowledged. First, as mentioned above, this study did not consider the possible influences affected by population density, influenza effectiveness, festival effects and other factors directly in the process of constructing the spatio-temporal routes. Although we have adopted the VARMA model which specifically dealt with residual effects as a remedy, it was definitely not as good as incorporating these factors into the analysis directly. Second, this study only analyzed the possible spreading directions of influenza between different cities from the perspective of spatio-temporal statistics. However, statistical significance cannot fully represent actual significance after all. In order to further demonstrate the issue of the spreading directions of spatio-temporal transmission, much additional work involving pathogenic detections, epidemiological investigations and so on still needs to be done. To this end, it is highly expected that this study could provide a bit inspiration and reference for future researches on surveillance of infectious diseases including but not limited to influenza.

## Conclusions

This study proposed a new framework for exploring the potentially stable spatio-temporal routes among different places and measuring specific sizes of the transmission effects. It showed that the period of influenza transmission cycle was longer in the Western Sichuan and Chengdu Plain than that in Northeastern Sichuan, and there would be potential spatio-temporal routes of influenza from bordering provinces or municipalities into Sichuan province. Furthermore, this study also pointed out several estimated spatio-temporal routes with relatively high strengths of associations, which could serve as clues of hot spot areas detection for influenza surveillance. The results could be used for the detection and early warning of infectious diseases in the future.

## Supplementary Information

**Additional file 1.** The association between traffic pattern and the spatio-temporal routes of Sichuan

**Additional file 2.** The parameter learning results using VARMA

## Data Availability

The data that support the findings of this study was obtained by applying it to the Sichuan CDC.

## Abbreviations

ILI: Influenza-like illness
DBN: Dynamic Bayesian network
VARMA: Vector autoregressive moving average
CDC: Center for Disease Control and Prevention
DAG: Directed acyclic graph
MTS: Multivariate time series
VAR: Vector-autoregressive model
CRAN: Comprehensive R Archive Network
