# Supplementary material for: Spatial transmission network construction of influenza-like illness using dynamic Bayesian network and vector-autoregressive moving average model
Source: BMC Infect Dis. 2021 Feb 10;21:164. doi: 10.1186/s12879-021-05769-6 (PMC7874476; doi:10.1186/s12879-021-05769-6)
Supplement: Supplementary file 1 — Additional file 1. The association between traffic pattern and the spatio-temporal routes of Sichuan [file 12879_2021_5769_MOESM1_ESM.docx]

**Additional file 1 The association between traffic pattern and the spatio-temporal routes of Sichuan**

In Chengdu Plain subregion, the Ziyang→Meishan route might originate from the Suizimei Expressway, and the Chengdu→Ya’an propagation route was to some extent in accord with the Chengya Expressway Direction; while the Guangyuan→Mianyang←→Deyang←→Chengdu→Ya’an propagation route was possibly in line with G5 Jingkun Expressway (the Mianguang section, the Chengmian section and the Chengya section), and the Nanchong ←→ Mianyang ←→ Deyang ←→ Chengdu route was expected to be brought by the Chengdemiannan Expressway. As another source, Leshan→Meishan←→Ya'an propagation route was guessed to be caused by Leya Expressway, which was another important highway flowing through Ya'an.

In Northeastern Sichuan subregion, Guangyuan was adjacent to both Shaanxi province and Gansu province, and National Highway 108 played an important channel for Shaanxi, Ningxia and Gansu entering Sichuan province. After the possible spreading of influenza from Guangyuan to Mianyang, another route from Mianyang to Nanchong was suspected to be spread via the Chengdemiannan Expressway. It was speculated that the propagation route from Nanchong to Suining and Dazhou was caused by National Highway 318 and the Suining-Nanchong and Suixi sections of G42 Rong Expressway.

In Southern Sichuan subregion, the main transmission routes were Yibin→Luzhou, Yibin→Leshan→Neijiang, and Yibin→Leshan←→Zigong→Neijiang. As was commonly known, Yibin played an essential transportation hub in southern Sichuan transportation network, and this city was a junction of Sichuan, Yunnan and Guizhou provinces with S206 provincial road crossing it. Therefore, it could be guessed that Yibin was one of the origins of influenza-like cases in Sichuan province, and the source was mainly from Yunnan. The main highways include Neiyi Expressway, Leyi Expressway and Yilu Expressway might explain why there were propagation routes between Yibin, Leshan and Luzhou. The propagation route between Leshan and Meishan was guessed by Chengle Expressway and Lemei Expressway; the route between Leshan and Yibin might be brought by Leyi Expressway; the propagation route between Leshan and Zigong might be brought by Lezi Expressway. The propagation route between Zigong and Neijiang should be formed by Neizi Expressway, Chengzilu and Zilong highway.

In Western Sichuan subregion, there existed G5 Beijing-Kunming Expressway across Panzhihua as a junction between Sichuan and Yunnan, and this expressway could be regarded as an important entrance from Yunnan to Sichuan. In terms of the spreading route from Panzhihua to Liangshan, G5 Beijing-Kunming Expressway was guessed to play an essential role.
