# Supplementary material for: Spatial transmission network construction of influenza-like illness using dynamic Bayesian network and vector-autoregressive moving average model
Source: BMC Infect Dis. 2021 Feb 10;21:164. doi: 10.1186/s12879-021-05769-6 (PMC7874476; doi:10.1186/s12879-021-05769-6)
Supplement: Supplementary file 2 — Additional file 2. The parameter learning results using VARMA [file 12879_2021_5769_MOESM2_ESM.docx]

**Additional file 2 The parameter learning results using VARMA**

**1) Aba, Chengdu, Deyang parameter** **learning results:**

**Table A1 the unit-root test result of Aba, Chengdu and Deyang**

| **lnILI** | **Dickey-Fuller** | ***P* value** |
| --- | --- | --- |
| Aba | -2.6065 | 0.3209 |
| Chengdu | -4.5264 | ＜0.01 |
| Deyang | -4.2371 | ＜0.01 |

The lnILI series in Aba State was not stable, so cointegration test was conducted for detecting cointegration relationship between those series. The result showed that cointegration relationship existed between them, and VARMA model could be fitted.

**Table A2 the two-way *p* value table for extended cross-correlation matrix of Aba, Chengdu and Deyang**

| **AR order: *p*** | **MA Order: *q*** | | | |
| --- | --- | --- | --- | --- |
|  | **0** | **1** | **2** | **3** |
| **0** | 0.0000 | 0.0000 | 0.0000 | 0.0000 |
| **1** | 0.0000 | 0.0075 | 0.1312 | 0.0765 |
| **2** | 0.5427 | 0.3264 | 0.6331 | 0.4455 |
| **3** | 0.9180 | 0.1500 | 0.7300 | 0.5614 |

As can be seen from table, the best order was *p*=2 and *q*=0, so we chose VARMA (2,0) for model fitting.

**Table A3 VARMA (2,0) first-order AR matrix of Aba, Chengdu, and Deyang**

| **Dependent variables** | **Independent variables** | | |
| --- | --- | --- | --- |
|  | **Aba** | **Chengdu** | **Deyang** |
| **Aba** | 0.37 | — | — |
| **Chengdu** | — | 1.40 | 0.14 |
| **Deyang** | — | -0.02 | 0.56 |

**Table A4 VARMA (2,0) second-order AR matrix of Aba, Chengdu and Deyang**

| **Dependent variables** | **Independent variables** | | |
| --- | --- | --- | --- |
|  | **Aba** | **Chengdu** | **Deyang** |
| **Aba** | 0.32 | — | — |
| **Chengdu** | — | 0.19 | -0.05 |
| **Deyang** | — | 0.17 | 0.07 |

**2) Aba, Chengdu, Ya'an parameter learning results:**

**Table A5 the unit-root test result of Aba, Chengdu and Ya'an**

| **lnILI** | **Dickey-Fuller** | ***P* value** |
| --- | --- | --- |
| **Aba** | -2.6065 | 0.3209 |
| **Chengdu** | -4.5264 | ＜0.01 |
| **Yaan** | -5.1313 | ＜0.01 |

The lnILI series in Aba State was not stable, so cointegration test was conducted for detecting cointegration relationship between those series. The result showed that cointegration relationship existed between them, and VARMA model could be fitted.

**Table A6 the two-way *p* value table for extended cross-correlation matrix of Aba, Chengdu and Deyang**

| **AR order: *p*** | **MA Order: *q*** | | | |
| --- | --- | --- | --- | --- |
|  | **0** | **1** | **2** | **3** |
| 0 | 0.0000 | 0.0000 | 0.0000 | 0.0000 |
| 1 | 0.0000 | 0.0004 | 0.2514 | 0.1337 |
| 2 | 0.0136 | 0.8519 | 0.9874 | 0.9270 |
| 3 | 0.9271 | 0.5223 | 0.9994 | 0.7345 |

As can be seen from table, the best order was *p*=2 and *q*=1, so we chose VARMA (2,1) for model fitting.

**Table A7** **VARMA (2,1) first-order AR matrix of Aba, Chengdu and Ya'an**

| **Dependent variables** | **Independent variables** | | |
| --- | --- | --- | --- |
|  | **Aba** | **Chengdu** | **Ya’an** |
| Aba | 0.28 | — | -0.67 |
| Chengdu | — | 1.40 | — |
| Ya’an | 0.13 | -0.27 | 1.07 |

**Table A8 VARMA (2,1) second-order AR matrix of Aba, Chengdu and Ya'an**

| **Dependent variables** | **Independent variables** | | |
| --- | --- | --- | --- |
|  | **Aba** | **Chengdu** | **Ya’an** |
| **Aba** | 0.32 | — | 0.13 |
| **Chengdu** | — | -0.42 | — |
| **Ya’an** | -0.10 | 0.24 | -0.13 |

**Table A9 VARMA (2,1) first-order MA matrix of Aba, Chengdu and Ya'an**

| **Dependent variables** | **Independent variables** | | |
| --- | --- | --- | --- |
|  | **Aba** | **Chengdu** | **Ya’an** |
| **Aba** | -0.06 | — | -0.70 |
| **Chengdu** | — | 0.65 | — |
| **Ya’an** | 0.12 | -0.37 | 0.67 |

**3) Aba, Deyang, Mianyang parameter learning results:**

**Table A10 the unit-root test result of Aba, Deyang and Mianyang**

| **lnILI** | **Dickey-Fuller** | ***P* value** |
| --- | --- | --- |
| **Aba** | -2.6065 | 0.3209 |
| **Deyang** | -4.2371 | ＜0.01 |
| **Mianyang** | -3.5827 | 0.0348 |

The lnILI series in Aba State was not stable, so cointegration test was conducted for detecting cointegration relationship between those series. The result showed that cointegration relationship existed between them, and VARMA model could be fitted.

**Table A11 the two-way *p* value table for extended cross-correlation matrix of Aba, Chengdu and Deyang**

| **AR order: *p*** | **MA Order: *q*** | | | |
| --- | --- | --- | --- | --- |
|  | **0** | **1** | **2** | **3** |
| **0** | 0.0000 | 0.0000 | 0.0000 | 0.0000 |
| **1** | 0.0000 | 0.1794 | 0.7098 | 0.5608 |
| **2** | 0.1701 | 0.6182 | 0.7775 | 0.7514 |
| **3** | 0.8067 | 0.9162 | 0.9942 | 0.8654 |

As can be seen from table, the best order was *p*=2 and *q*=0, so we chose VARMA (2,0) for model fitting.

**Table A12 VARMA (2,0) first-order AR matrix of Aba, Deyang and Mianyang**

| **Dependent variables** | **Independent variables** | | |
| --- | --- | --- | --- |
|  | **Aba** | **Deyang** | **Mianyang** |
| **Aba** | 0.38 | — | — |
| **Deyang** | — | 0.61 | -0.14 |
| **Mianyang** | — | 0.00 | 0.64 |

**Table A13 VARMA (2,0) second-order AR matrix Aba, Deyang and Mianyang**

| **Dependent variables** | **Independent variables** | | |
| --- | --- | --- | --- |
|  | **Aba** | **Deyang** | **Mianyang** |
| **Aba** | 0.32 | — | — |
| **Deyang** | — | 0.13 | 0.06 |
| **Mianyang** | — | -0.07 | 0.26 |

**4) Aba, Ganzi, Ya'an parameter learning results:**

**Table A14 the unit-root test result of Aba, Ganzi and Ya'an**

| **lnILI** | **Dickey-Fuller** | ***P* value** |
| --- | --- | --- |
| **Aba** | -2.6231 | 0.3139 |
| **Ganzi** | -5.9420 | ＜0.01 |
| **Ya’an** | -5.1363 | ＜0.01 |

The lnILI series in Aba State was not stable, so cointegration test was conducted for detecting cointegration relationship between those series. The result showed that cointegration relationship existed between them, and VARMA model could be fitted.

**Table A15 the two-way *p* value table for extended cross-correlation matrix of Aba, Ganzi and Ya'an**

| **AR order: *p*** | **MA Order: *q*** | | | |
| --- | --- | --- | --- | --- |
|  | **0** | **1** | **2** | **3** |
| **0** | 0.0000 | 0.0000 | 0.0000 | 0.0000 |
| **1** | 0.0000 | 0.0001 | 0.0185 | 0.1216 |
| **2** | 0.0003 | 0.0293 | 0.7671 | 0.9222 |
| **3** | 0.4486 | 0.9498 | 0.9971 | 0.9989 |

As can be seen from table, the best order was *p*=3 and *q*=0, so we chose VARMA (3,0) for model fitting.

**Table A16 VARMA (3,0) first-order AR matrix of Aba, Ganzi and Ya'an**

| **Dependent variables** | **Independent variables** | | |
| --- | --- | --- | --- |
|  | **Aba** | **Ganzi** | **Ya’an** |
| **Aba** | 0.37 | — | 0.06 |
| **Ganzi** | — | 0.38 | — |
| **Ya’an** | 0.02 | — | 0.48 |

**Table A17 VARMA (3,0) second-order AR matrix of Aba, Ganzi and Ya'an**

| **Dependent variables** | **Independent variables** | | |
| --- | --- | --- | --- |
|  | **Aba** | **Ganzi** | **Ya’an** |
| **Aba** | 0.30 | — | -0.19 |
| **Ganzi** | — | 0.17 | — |
| **Ya’an** | -0.04 | — | 0.04 |

**Table A18 VARMA (3,0) third-order AR matrix of Aba, Ganzi and Ya'an**

| **Dependent variables** | **Independent variables** | | |
| --- | --- | --- | --- |
|  | **Aba** | **Ganzi** | **Ya’an** |
| **Aba** | 0.04 | — | 0.02 |
| **Ganzi** | — | 0.07 | — |
| **Ya’an** | 0.00 | — | 0.26 |

**5) Bazhong, Dazhou, Nanchong parameters learning results:**

**Table A19 the unit-root test result of** **Bazhong, Dazhou and Nanchong**

| **lnILI** | **Dickey-Fuller** | ***P* value** |
| --- | --- | --- |
| **Bazhong** | -3.8706 | 0.0160 |
| **Dazhou** | -2.0552 | 0.5526 |
| **Nanchong** | -3.3749 | 0.0591 |

The lnILI series in Dazhou and Nanchong was not stable, so cointegration test was conducted for detecting cointegration relationship between those series. The result showed that cointegration relationship existed between them, and VARMA model could be fitted.

**Table A20 the two-way *p* value table for extended cross-correlation matrix of Bazhong, Dazhou and Nanchong**

| **AR order: *p*** | **MA Order: *q*** | | | |
| --- | --- | --- | --- | --- |
|  | **0** | **1** | **2** | **3** |
| **0** | 0.0000 | 0.0000 | 0.0000 | 0.0000 |
| **1** | 0.0000 | 1.0000 | 0.8371 | 0.5473 |
| **2** | 0.0312 | 0.9879 | 0.8658 | 0.9983 |
| **3** | 0.9281 | 1.0000 | 1.0000 | 0.9959 |

As can be seen from the table, the best order was *p*=1 and *q*=1, we chose VARMA (1,1) for model fitting.

**Table A21 VARMA (1,1) first-order AR matrix of Bazhong, Dazhou and Nanchong**

| **Dependent variables** | **Independent variables** | | |
| --- | --- | --- | --- |
|  | **Bazhong** | **Dazhou** | **Nanchong** |
| **Bazhong** | -0.67 | — | — |
| **Dazhou** | — | 1.42 | -0.11 |
| **Nanchong** | — | — | 0.69 |

**Table A22 VARMA (1,1) first-order MA matrix of Bazhong, Dazhou and Nanchong**

| **Dependent variables** | **Independent variables** | | |
| --- | --- | --- | --- |
|  | **Bazhong** | **Dazhou** | **Nanchong** |
| **Bazhong** | -0.65 | — | — |
| **Dazhou** | — | 1.38 | -0.28 |
| **Nanchong** | — | — | 0.25 |

**6) Bazhong, Guangyuan, Nanchong parameters learning results:**

**Table A23 the unit-root test result of Bazhong, Guangyuan and Nanchong**

| **lnILI** | **Dickey-Fuller** | ***P* value** |
| --- | --- | --- |
| **Bazhong** | -3.8706 | 0.0160 |
| **Guangyuan** | -4.0131 | ＜0.01 |
| **Nanchong** | -3.3749 | 0.0591 |

The lnILI series in Nanchong city was not stable, so cointegration test was conducted for detecting cointegration relationship between those series. The result showed that cointegration relationship existed between them, and VARMA model could be fitted.

**Table A24 the two-way *p* value table for extended cross-correlation matrix of Bazhong, Guangyuan and Nanchong**

| **AR order: *p*** | **MA Order: *q*** | | | |
| --- | --- | --- | --- | --- |
|  | **0** | **1** | **2** | **3** |
| **0** | 0.0000 | 0.0000 | 0.0000 | 0.0000 |
| **1** | 0.0009 | 0.8735 | 0.7776 | 0.6722 |
| **2** | 0.3426 | 0.8536 | 0.9832 | 0.9522 |
| **3** | 0.9567 | 0.9946 | 1.0000 | 0.9969 |

As can be seen from the table, the best order was *p*=2 and *q*=0, so we chose VARMA (2,0) for model fitting.

**Table A25 VARMA (2,0) first-order AR matrix of Bazhong, Guangyuan and Nanchong**

| **Dependent variables** | **Independent variables** | | |
| --- | --- | --- | --- |
|  | **Bazhong** | **Guangyuan** | **Nanchong** |
| **Bazhong** | -0.60 | — | — |
| **Guangyuan** | — | 0.77 | — |
| **Nanchong** | — | — | 1.09 |

**Table A26 VARMA (2,0) second-order AR matrix of Bazhong, Guangyuan and Nanchong**

| **Dependent variables** | **Independent variables** | | |
| --- | --- | --- | --- |
|  | **Bazhong** | **Guangyuan** | **Nanchong** |
| **Bazhong** | -0.60 | — | — |
| **Guangyuan** | — | 0.33 | — |
| **Nanchong** | — | — | 0.55 |

**7) Chengdu, Deyang, Ziyang parameters learning results:**

**Table A27 the unit-root test result of Chengdu, Deyang and Ziyang**

| **lnILI** | **Dickey-Fuller** | ***P* value** |
| --- | --- | --- |
| Chengdu | -4.5264 | ＜0.01 |
| Deyang | -4.2371 | ＜0.01 |
| Ziyang | -1.9973 | 0.5781 |

The lnILI series in Ziyang State was not stable, so cointegration test was conducted for detecting cointegration relationship between those series. The result showed that cointegration relationship existed between them, and VARMA model could be fitted.

**Table A28 the two-way *p* value table for extended cross-correlation matrices of Chengdu, Deyang and Ziyang**

| **AR order: *p*** | **MA Order: *q*** | | | |
| --- | --- | --- | --- | --- |
|  | **0** | **1** | **2** | **3** |
| **0** | 0.0000 | 0.0000 | 0.0000 | 0.0000 |
| **1** | 0.0000 | 0.1243 | 0.4038 | 0.4328 |
| **2** | 0.6152 | 0.7812 | 0.9953 | 0.7315 |
| **3** | 0.9996 | 0.9851 | 0.9981 | 0.8898 |

As can be seen from the table, the best order was *p*=2 and *q*=0, so we chose VARMA (2,0) for model fitting.

**Table A29 VARMA (2,0) first-order AR matrix of Chengdu, Deyang and Ziyang**

| **Dependent variables** | **Independent variables** | | |
| --- | --- | --- | --- |
|  | **Chengdu** | **Deyang** | **Ziyang** |
| **Chengdu** | 0.67 | 0.14 | — |
| **Deyang** | -0.02 | 0.56 | — |
| **Ziyang** | — | — | 0.55 |

**Table** **A30 VARMA (2,0) second-order AR matrix of Chengdu, Deyang and Ziyang**

| **Dependent variables** | **Independent variables** | | |
| --- | --- | --- | --- |
|  | **Chengdu** | **Deyang** | **Ziyang** |
| **Chengdu** | 0.20 | -0.04 | — |
| **Deyang** | 0.16 | 0.07 | — |
| **Ziyang** | — | — | 0.40 |

**8) Chengdu, Meishan, Ya'an parameter learning results:**

**Table A31 the unit-root test result of Chengdu, Meishan and Ya'an**

| **lnILI** | **Dickey-Fuller** | ***P* value** |
| --- | --- | --- |
| **Chengdu** | -4.5264 | ＜0.01 |
| **Meishan** | -4.4232 | ＜0.01 |
| **Ya’an** | -5.1313 | ＜0.01 |

It showed that the lnILI series of Chengdu, Meishan City and Ya'an were all stable, and the VARMA model can be fitted.

**Table A32 the two-way *p* value table for extended cross-correlation matrices of Chengdu, Meishan and Ya'an**

| **AR order: *p*** | **MA Order: *q*** | | | |
| --- | --- | --- | --- | --- |
|  | **0** | **1** | **2** | **3** |
| **0** | 0.0000 | 0.0000 | 0.0000 | 0.0000 |
| **1** | 0.0000 | 0.1243 | 0.4038 | 0.4328 |
| **2** | 0.6152 | 0.7812 | 0.9953 | 0.7315 |
| **3** | 0.9996 | 0.9851 | 0.9981 | 0.8898 |

As can be seen from the table, the best order was *p*=2 and *q*=1, so we chose VARMA (2,1) for model fitting.

**Table A33 VARMA (2,1) first-order AR matrix of Chengdu, Meishan and Ya'an**

| **Dependent variables** | **Independent variables** | | |
| --- | --- | --- | --- |
|  | **Chengdu** | **Meishan** | **Ya’an** |
| **Chengdu** | 0.81 | — | — |
| **Meishan** | — | 1.11 | -0.13 |
| **Ya’an** | -0.38 | — | 1.06 |

**Table A34 VARMA (2,1) second-order AR matrix of Chengdu, Meishan and Ya'an**

| **Dependent variables** | **Independent variables** | | |
| --- | --- | --- | --- |
|  | **Chengdu** | **Meishan** | **Ya’an** |
| **Chengdu** | 0.12 | — | — |
| **Meishan** | — | -0.08 | -0.03 |
| **Ya’an** | 0.34 | — | -0.18 |

**Table A35 VARMA (2,1) first-order MA matrix of Chengdu, Meishan and Ya'an**

| **Dependent variables** | **Independent variables** | | |
| --- | --- | --- | --- |
|  | **Chengdu** | **Meishan** | **Ya’an** |
| **Chengdu** | 0.12 | — | — |
| **Meishan** | — | 0.86 | -0.38 |
| **Ya’an** | 0.34 | — | -0.18 |

**9) Chengdu, Meishan, Ziyang parameters learning results:**

**Table A36 the unit-root test result of Chengdu, Meishan and Ziyang**

| **lnILI** | **Dickey-Fuller** | ***P* value** |
| --- | --- | --- |
| **Chengdu** | -4.5264 | ＜0.01 |
| **Meishan** | -4.4232 | ＜0.01 |
| **Ziyang** | -1.9973 | 0.5781 |

The lnILI series in Ziyang State was not stable, so cointegration test was conducted for detecting cointegration relationship between those series. The result showed that cointegration relationship existed between them, and VARMA model could be fitted.

**Table A37 the two-way *p* value table for extended cross-correlation matrix of Chengdu, Meishan and Ziyang**

| **AR order: *p*** | **MA Order: *q*** | | | |
| --- | --- | --- | --- | --- |
|  | **0** | **1** | **2** | **3** |
| **0** | 0.0000 | 0.0000 | 0.0000 | 0.0000 |
| **1** | 0.0000 | 0.2949 | 0.7846 | 0.4411 |
| **2** | 0.7353 | 0.9509 | 0.9575 | 0.5817 |
| **3** | 0.9989 | 0.9969 | 0.9331 | 0.7844 |

As can be seen from the table, the best order was *p*=2 and *q*=0, so we chose VARMA (2,0) for model fitting.

**Table A38 VARMA (2,0) first-order AR matrix of Chengdu, Meishan and Ziyang**

| **Dependent variables** | **Independent variables** | | |
| --- | --- | --- | --- |
|  | **Chengdu** | **Meishan** | **Ziyang** |
| **Chengdu** | 0.70 | — | — |
| **Meishan** | — | 0.27 | 0.35 |
| **Ziyang** | — | — | 0.55 |

**Table A39 VARMA (2,0) second-order AR matrix of Chengdu, Meishan and Ziyang**

| **Dependent variables** | **Independent variables** | | |
| --- | --- | --- | --- |
|  | **Chengdu** | **Meishan** | **Ziyang** |
| **Chengdu** | 0.21 | — | — |
| **Meishan** | — | 0.17 | -0.09 |
| **Ziyang** | — | — | 0.39 |

**10) Dazhou, Guang'an, Nanchong parameter learning results:**

**Table A40 the unit-root test result of Dazhou, Guang'an and Nanchong**

| **lnILI** | **Dickey-Fuller** | ***P* value** |
| --- | --- | --- |
| **Dazhou** | -2.0433 | 0.5576 |
| **Guang'an** | -1.5254 | 0.7757 |
| **Nanchong** | -3.4922 | 0.0440 |

The lnILI series in Dazhou and Guang’an was not stable, so cointegration test was conducted for detecting cointegration relationship between those series. The result showed that cointegration relationship existed between them, and VARMA model could be fitted.

**Table A41 the two-way *p* value table for extended cross-correlation matrices of Dazhou, Guang'an and Nanchong**

| **AR order: *p*** | **MA Order: *q*** | | | |
| --- | --- | --- | --- | --- |
|  | **0** | **1** | **2** | **3** |
| **0** | 0.0000 | 0.0000 | 0.0000 | 0.0000 |
| **1** | 0.0001 | 0.6825 | 0.5446 | 0.2866 |
| **2** | 0.1836 | 0.5636 | 0.0976 | 0.0092 |
| **3** | 0.9899 | 0.9619 | 0.1191 | 0.9117 |

As can be seen from the table, the best order was *p*=1 and *q*=1, so we chose VARMA (1,1) for model fitting.

**Table A42 VARMA (1,1) first-order AR matrix of Dazhou, Guang'an and Nanchong**

| **Dependent variables** | **Independent variables** | | |
| --- | --- | --- | --- |
|  | **Dazhou** | **Guang'an** | **Nanchong** |
| **Dazhou** | 0.81 | — | -0.23 |
| **Guang'an** | — | 0.84 | — |
| **Nanchong** | — | — | 0.75 |

**Table A43 VARMA (1,1) first-order MA matrix of Dazhou, Guang'an and Nanchong**

| **Dependent variables** | **Independent variables** | | |
| --- | --- | --- | --- |
|  | **Dazhou** | **Guang'an** | **Nanchong** |
| **Dazhou** | 0.67 | — | -0.23 |
| **Guang'an** | — | 0.20 | — |
| **Nanchong** | — | — | 0.26 |

**11) Deyang, Mianyang, Suining parameters learning results:**

**Table A44 the unit-root test result of** **Deyang, Mianyang and Suining**

| **lnILI** | **Dickey-Fuller** | ***P* value** |
| --- | --- | --- |
| **Deyang** | -4.2371 | ＜0.01 |
| **Mianyang** | -3.5827 | 0.03488 |
| **Suining** | -4.1879 | ＜0.01 |

It showed that series of Deyang, Mianyang and Suining were all stable, so we could fit the VARMA model.

**Table A45 the two-way *p* value table for extended cross-correlation matrix of Deyang, Mianyang and Suining**

| **AR order: *p*** | **MA Order: *q*** | | | |
| --- | --- | --- | --- | --- |
|  | **0** | **1** | **2** | **3** |
| **0** | 0.0000 | 0.0000 | 0.0000 | 0.0000 |
| **1** | 0.0000 | 0.1281 | 0.2074 | 0.2785 |
| **2** | 0.6434 | 0.3446 | 0.8208 | 0.7008 |
| **3** | 0.9980 | 0.9825 | 0.9990 | 0.9859 |

As can be seen from the table, the best order was *p*=2 and *q*=0, so we chose VARMA (2,0) for model fitting.

**Table A46 VARMA (2,0) first-order AR matrix of Deyang, Mianyang and Suining**

| **Dependent variables** | **Independent variables** | | |
| --- | --- | --- | --- |
|  | **Deyang** | **Mianyang** | **Suining** |
| **Deyang** | 0.61 | -0.14 | — |
| **Mianyang** | 0.00 | 0.64 | — |
| **Suining** | — | — | 1.06 |

**Table A47 VARMA (2,0) second-order AR matrix of Deyang, Mianyang and Suining**

| **Dependent variables** | **Independent variables** | | |
| --- | --- | --- | --- |
|  | **Deyang** | **Mianyang** | **Suining** |
| **Deyang** | 0.13 | 0.05 | — |
| **Mianyang** | -0.08 | 0.26 | — |
| **Suining** | — | — | 0.25 |

**12) Ganzi, Liangshan, Ya'an parameter learning results:**

**Table A48 the unit-root test results of** **Ganzi, Liangshan and Ya'an**

| **lnILI** | **Dickey-Fuller** | ***P* value** |
| --- | --- | --- |
| **Ganzi** | -5.3029 | ＜0.01 |
| **Liangshan** | -4.6364 | ＜0.01 |
| **Ya’an** | -3.9003 | 0.0149 |

It showed that the lnILI series in Ganzi, Liangshan and Ya'an were all stable, and the VARMA model could be fitted.

**Table A49 the two-way *p* value table for extended cross-correlation matrix of Ganzi, Liangshan and Ya'an**

| **AR order: *p*** | **MA Order: *q*** | | | |
| --- | --- | --- | --- | --- |
|  | **0** | **1** | **2** | **3** |
| **0** | 0.0000 | 0.0000 | 0.0000 | 0.0000 |
| **1** | 0.0000 | 0.0303 | 0.0075 | 0.0953 |
| **2** | 0.0184 | 0.1500 | 0.9759 | 0.8373 |
| **3** | 0.6108 | 0.9109 | 0.9968 | 0.8710 |

As can be seen from the table, the best order was *p*=3 and *q*=0, so we chose VARMA (3,0) for model fitting.

**Table A50 VARMA (3,0) first-order AR matrix of Ganzi, Liangshan and Ya'an**

| **Dependent variables** | **Independent variables** | | |
| --- | --- | --- | --- |
|  | **Ganzi** | **Liangshan** | **Ya‘’an** |
| Ganzi | 0.42 | — | — |
| Liangshan | — | 0.06 | — |
| Ya’an | — | — | 0.51 |

**Table A51 VARMA (3,0) second-order AR matrix of Ganzi, Liangshan and Ya'an**

| **Dependent variables** | **Independent variables** | | |
| --- | --- | --- | --- |
|  | **Ganzi** | **Liangshan** | **Ya’an** |
| **Ganzi** | 0.15 | — | — |
| **Liangshan** | — | 0.10 | — |
| **Ya‘’an** | — | — | 0.10 |

**Table A52 VARMA (3,0) third-order AR matrix of Ganzi, Liangshan and Ya'an**

| **Dependent variables** | **Independent variables** | | |
| --- | --- | --- | --- |
|  | **Ganzi** | **Liangshan** | **Ya’an** |
| **Ganzi** | 0.08 | — | — |
| **Liangshan** | — | 0.12 | — |
| **Ya’an** | — | — | 0.21 |

**13) Guang'an, Nanchong, Suining parameters learning results:**

**Table A53 the unit-root test results of Guang'an, Nanchong and Suining**

| **lnILI** | **Dickey-Fuller** | ***P* value** |
| --- | --- | --- |
| **Guang'an** | -1.5254 | 0.7757 |
| **Nanchong** | -3.4922 | 0.044 |
| **Suining** | -4.2645 | ＜0.01 |

The lnILI series in Guang’an was not stable, so cointegration test was conducted for detecting cointegration relationship between those series. The result showed that cointegration relationship existed between them, and VARMA model could be fitted.

**Table A54 The two-way *p* value table for extended cross-correlation matrices of Guang'an, Nanchong and Suining**

| **AR order: *p*** | **MA Order: *q*** | | | |
| --- | --- | --- | --- | --- |
|  | **0** | **1** | **2** | **3** |
| **0** | 0.0000 | 0.0000 | 0.0000 | 0.0000 |
| **1** | 0.0127 | 0.5098 | 0.2165 | 0.1883 |
| **2** | 0.3647 | 0.6783 | 0.0860 | 0.0353 |
| **3** | 0.9956 | 0.8249 | 0.2915 | 0.6159 |

As can be seen from the table, the best order was *p*=1 and *q*=1, so we chose VARMA (1,1) for model fitting.

**Table A55 VARMA (1,1) first-order AR matrix of Guang'an, Nancheng and Suining**

| **Dependent variables** | **Independent variables** | | |
| --- | --- | --- | --- |
|  | **Guang'an** | **Nanchong** | **Suining** |
| **Guang'an** | 0.95 | — | — |
| **Nanchong** | — | 0.98 | — |
| **Suining** | — | -0.01 | 0.77 |

Ta**ble A56 VARMA (1,1) first-order MA matrix of Guang'an, Nancheng and Suining**

| **Dependent variables** | **Independent variables** | | |
| --- | --- | --- | --- |
|  | **Guang'an** | **Nanchong** | **Suining** |
| **Guang'an** | 0.39 | — | — |
| **Nanchong** | — | 0.48 | — |
| **Suining** | — | -0.17 | 0.27 |

**14) Guangyuan, Mianyang, Nanchong parameters learning results:**

**Table A57 the unit-root test result of** **Guangdong, Mianyang and Nanchong**

| **lnILI** | **Dickey-Fuller** | ***P* value** |
| --- | --- | --- |
| **Guangyuan** | -4.0217 | ＜0.01 |
| **Mianyang** | -4.3665 | ＜0.01 |
| **Nanchong** | -3.4922 | 0.044 |

It showed that the lnILI series of Guangyuan City, Mianyang City and Nanchong City were all stable, so the VARMA model could be fitted.

**Table A58 the two-way *p* value table for extended cross-correlation matrix of Guangdong, Mianyang and Nanchong**

| **AR order: *p*** | **MA Order: *q*** | | | |
| --- | --- | --- | --- | --- |
|  | **0** | **1** | **2** | **3** |
| **0** | 0.0000 | 0.0000 | 0.0000 | 0.0000 |
| **1** | 0.0027 | 0.3433 | 0.5525 | 0.9628 |
| **2** | 0.5331 | 0.2360 | 0.6965 | 0.1915 |
| **3** | 0.9749 | 0.8600 | 0.7414 | 0.9829 |

As can be seen from the table, the best order was *p*=1 and *q*=1, so we chose VARMA (1,1) for model fitting.

**Table A59 VARMA (1,1) first-order AR matrix of Guangyuan, Mianyang and Nanchong**

| **Dependent variables** | **Independent variables** | | |
| --- | --- | --- | --- |
|  | **Guangyuan** | **Mianyang** | **Nanchong** |
| **Guangyuan** | 0.83 | — | — |
| **Mianyang** | -0.07 | 0.97 | 0.00 |
| **Nanchong** | — | 0.09 | 0.69 |

**Table A60 VARMA (1,1) first-order MA matrix of Guangyuan, Mianyang and Nanchong**

| **Dependent variables** | **Independent variables** | | |
| --- | --- | --- | --- |
|  | **Guangyuan** | **Mianyang** | **Nanchong** |
| **Guangyuan** | 0.36 | — | — |
| **Mianyang** | 0.02 | 0.19 | -0.21 |
| **Nanchong** | — | -0.09 | 0.16 |

**15) Leshan, Liangshan, Meishan, Ya'an parameters learning results:**

**Table A61 the unit-root test results of Leshan, Liangshan, Meishan and Ya'an**

| **lnILI** | **Dickey-Fuller** | ***P* value** |
| --- | --- | --- |
| **Leshan** | -3.7665 | 0.02157 |
| **Liangshan** | -4.6419 | ＜0.01 |
| **Meishan** | -4.3098 | ＜0.01 |
| **Ya’an** | -3.8937 | 0.01525 |

It showed that lnILI series of Leshan, Liangshan, Meishan and Ya'an were all stable, then we could fit the VARMA model.

**Table A62 the two-way *p* value table for extended cross-correlation matrix of Aba, Chengdu and Deyang**

| **AR order: *p*** | **MA Order: *q*** | | | |
| --- | --- | --- | --- | --- |
|  | **0** | **1** | **2** | **3** |
| **0** | 0.0000 | 0.0000 | 0.0000 | 0.0000 |
| **1** | 0.0000 | 0.0117 | 0.0747 | 0.4384 |
| **2** | 0.0982 | 0.0783 | 0.9994 | 0.9733 |
| **3** | 0.9716 | 1.0000 | 1.0000 | 1.0000 |

As can be seen from the table, the best order was *p*=3 and *q*=0, so we chose VARMA (3,0) for model fitting.

**Table A63 VARMA (3,0) first-order AR matrix of Leshan, Liangshan, Meishan and Ya'an**

| **Dependent variables** | **Independent variables** | | | |
| --- | --- | --- | --- | --- |
|  | **Leshan** | **Liangshan** | **Meishan** | **Ya’an** |
| **Leshan** | 0.46 | 0.04 | — | — |
| **Liangshan** | 0.75 | 0.07 | — | — |
| **Meishan** | -0.83 | — | 0.35 | 0.53 |
| **Ya’an** | — | — | 0.07 | 0.48 |

**Table A64 VARMA (3,0) second-order AR matrix of Leshan, Liangshan, Meishan and Ya'an**

| **Dependent variables** | **Independent variables** | | | |
| --- | --- | --- | --- | --- |
|  | **Leshan** | **Liangshan** | **Meishan** | **Ya’an** |
| **Leshan** | 0.28 | -0.05 | — | — |
| **Liangshan** | 0.00 | 0.11 | — | — |
| **Meishan** | 0.84 | — | 0.15 | -0.38 |
| **Ya’an** | — | — | -0.02 | 0.08 |

**Table A65 VARMA (3,0) third-order AR matrix of Leshan, Liangshan, Meishan and Ya'an**

| **Dependent variables** | **Independent variables** | | | |
| --- | --- | --- | --- | --- |
|  | **Leshan** | **Liangshan** | **Meishan** | **Ya’an** |
| **Leshan** | 0.17 | -0.01 | — | — |
| **Liangshan** | -0.90 | 0.12 | — | — |
| **Meishan** | -0.16 | — | 0.11 | 0.11 |
| **Ya’an** | — | — | -0.03 | 0.22 |

**16) Leshan, Liangshan, Yibin parameter learning results:**

**Table A66 the unit-root test results of Leshan, Liangshan and Yibin**

| **lnILI** | **Dickey-Fuller** | ***P* value** |
| --- | --- | --- |
| **Leshan** | -3.7429 | 0.02275 |
| **Liangshan** | -4.6393 | ＜0.01 |
| **Yibin** | -2.1366 | 0.5186 |

The lnILI series in Yibin was not stable, so cointegration test was conducted for detecting cointegration relationship between those series. The result showed that cointegration relationship existed between them, and VARMA model could be fitted.

**Table A67 the two-way *p* value table for extended cross-correlation matrix of Leshan, Liangshan and Yibin**

| **AR order: *p*** | **MA Order: *q*** | | | |
| --- | --- | --- | --- | --- |
|  | **0** | **1** | **2** | **3** |
| **0** | 0.0000 | 0.0000 | 0.0000 | 0.0000 |
| **1** | 0.0000 | 0.5923 | 0.7165 | 0.8453 |
| **2** | 0.1309 | 0.6622 | 0.9996 | 0.9999 |
| **3** | 0.8094 | 0.9998 | 1.0000 | 0.9992 |

As can be seen from the table, the best order was *p*=2 and *q*=0, so we chose VARMA(2,0) for model fitting.

**Table A68 VARMA (2,0) first-order AR matrix of Leshan, Liangshan and Yibin**

| **Dependent variables** | **Independent variables** | | |
| --- | --- | --- | --- |
|  | **Leshan** | **Liangshan** | **Yibin** |
| **Leshan** | 0.55 | 0.04 | -0.01 |
| **Liangshan** | 0.33 | 0.10 | — |
| **Yibin** | — | — | 0.21 |

**Table A69 VARMA (2,0) second-order AR matrix of Leshan, Liangshan and Yibin**

| **Dependent variables** | **Independent variables** | | |
| --- | --- | --- | --- |
|  | **Leshan** | **Liangshan** | **Yibin** |
| **Leshan** | 0.34 | -0.05 | -0.03 |
| **Liangshan** | -0.43 | 0.08 | — |
| **Yibin** | — | — | 0.15 |

**17) Leshan, Meishan, Neijiang, Zigong parameters learning results:**

**Table A70 the unit-root test result of Leshan, Meishan, Neijiang and Zigong**

| **lnILI** | **Dickey-Fuller** | ***P* value** |
| --- | --- | --- |
| **Leshan** | -4.9608 | ＜0.01 |
| **Meishan** | -4.4043 | ＜0.01 |
| **Neijiang** | -4.1211 | ＜0.01 |
| **Zigong** | -4.8550 | ＜0.01 |

It showed that the lnILI series of Leshan, Meishan, Neijiang and Zigong were all stable, and the VARMA model could be fitted.

**Table A71 the two-way *p* value table for extended cross-correlation matrix of Leshan, Meishan, Neijiang and Zigong**

| **AR order: *p*** | **MA Order: *q*** | | | |
| --- | --- | --- | --- | --- |
|  | **0** | **1** | **2** | **3** |
| **0** | 0.0000 | 0.0000 | 0.0000 | 0.0000 |
| **1** | 0.0000 | 0.2260 | 0.4223 | 0.2982 |
| **2** | 0.1989 | 0.5902 | 0.8778 | 0.9971 |
| **3** | 0.9075 | 0.9873 | 0.9944 | 0.8848 |

As can be seen from the table, the best order was *p*=1 and *q*=1, so we chose VARMA (1,1) for model fitting.

**Table A72 VARMA (1,1) first-order AR matrix of Leshan, Meishan, Neijiang and Zigong**

| **Dependent variables** | **Independent variables** | | | |
| --- | --- | --- | --- | --- |
|  | **Leshan** | **Meishan** | **Neijiang** | **Zigong** |
| **Leshan** | 0.90 | — | — | -0.02 |
| **Meishan** | -0.05 | 0.92 | — | — |
| **Neijiang** | -0.10 | — | 0.87 | — |
| **Zigong** | -0.09 | — | — | 0.88 |

T**able A73 VARMA (1,1) first-order MA matrix of Leshan, Meishan, Neijiang and Zigong**

| **Dependent variables** | **Independent variables** | | | |
| --- | --- | --- | --- | --- |
|  | **Leshan** | **Meishan** | **Neijiang** | **Zigong** |
| **Leshan** | 0.38 | — | — | -0.12 |
| **Meishan** | -0.02 | 0.75 | — | — |
| **Neijiang** | -0.11 | — | 0.61 | — |
| **Zigong** | -0.13 | — | — | 0.29 |

**18) Leshan, Yibin, Zigong parameters learning results:**

**Table A74 the unit-root test result of Leshan, Yibin and Zigong**

| **lnILI** | **Dickey-Fuller** | ***P* value** |
| --- | --- | --- |
| **Leshan** | -5.0156 | ＜0.01 |
| **Yibin** | -3.4497 | 0.0477 |
| **Zigong** | -4.8704 | ＜0.01 |

It showed that the lnILI series of Leshan, Yibin and Zigong were all stable, then we can fit the VARMA model.

**Table A75 the two-way *p* value table for extended cross-correlation matrix of Leshan, Yibin, and Zigong**

| **AR order: *p*** | **MA Order: *q*** | | | |
| --- | --- | --- | --- | --- |
|  | **0** | **1** | **2** | **3** |
| **0** | 0.0000 | 0.0000 | 0.0000 | 0.0000 |
| **1** | 0.0000 | 0.9526 | 0.9487 | 0.8085 |
| **2** | 0.1590 | 0.9955 | 0.9985 | 0.9997 |
| **3** | 0.8644 | 0.9842 | 0.9990 | 0.9799 |

As can be seen from the table, the best order was *p*=1 and *q*=1, so we fit VARMA (1,1) for model fitting:

**Table A76 VARMA (1,1) first-order AR matrix of Leshan, Yibin and Zigong**

| **Dependent variables** | **Independent variables** | | |
| --- | --- | --- | --- |
|  | **Leshan** | **Yibin** | **Zigong** |
| **Leshan** | 0.89 | — | -0.13 |
| **Yibin** | — | 0.80 | — |
| **Zigong** | -0.10 | — | 0.82 |

**Table A77 VARMA (1,1) first-order MA matrix of Leshan, Yibin and Zigong**

| **Dependent variables** | **Independent variables** | | |
| --- | --- | --- | --- |
|  | **Leshan** | **Yibin** | **Zigong** |
| **Leshan** | 0.36 | — | -0.23 |
| **Yibin** | — | 0.60 | — |
| **Zigong** | -0.14 | — | 0.20 |

**19)Liangshan and Panzhihua parameter learning results:**

**Table A78 the unit-root test result of Liangshan and Panzhihua**

| **lnILI** | **Dickey-Fuller** | ***P* value** |
| --- | --- | --- |
| **Liangshan** | -4.6419 | ＜0.01 |
| **Panzhihua** | -1.8954 | 0.6199 |

The lnILI series in Panzhihua was not stable, so cointegration test was conducted for detecting cointegration relationship between those series. The result showed that cointegration relationship existed between them, and VARMA model could be fitted.

**Table A2 the two-way *p* value table for extended cross-correlation matrix of Liangshan and Panzhihua**

| **AR order: *p*** | **MA Order: *q*** | | | |
| --- | --- | --- | --- | --- |
|  | **0** | **1** | **2** | **3** |
| **0** | 0.0000 | 0.0000 | 0.0000 | 0.0000 |
| **1** | 0.0000 | 0.9986 | 0.9769 | 0.7209 |
| **2** | 0.1968 | 0.9990 | 0.9641 | 0.7674 |
| **3** | 0.6353 | 0.9993 | 0.9845 | 0.7513 |

As can be seen from the table, the best order was *p*=2 and *q*=0, so we chose VARMA (2,0) for model fitting.

**Table A79 VARMA (2,0) first-order AR matrix of Liangshan and Panzhihua**

| **Dependent variables** | **Independent variables** | |
| --- | --- | --- |
|  | **Liangshan** | **Panzhihua** |
| **Liangshan** | 0.07 | 0.17 |
| **Panzhihua** | — | 0.65 |

**Table A80 VARMA (2,0) second-order AR matrix of Liangshan and Panzhihua**

| **Dependent variables** | **Independent variables** | |
| --- | --- | --- |
|  | **Liangshan** | **Panzhihua** |
| **Liangshan** | 0.10 | 0.00 |
| **Panzhihua** | — | 0.30 |

**20) Luzhou, Neijiang and Zigong parameter learning results:**

**Table A81 the unit-root test result of** **Luzhou, Neijiang and Zigong**

| **lnILI** | **Dickey-Fuller** | ***P* value** |
| --- | --- | --- |
| **Luzhou** | -4.8754 | ＜0.01 |
| **Neijiang** | -4.1211 | ＜0.01 |
| **Zigong** | -4.8550 | ＜0.01 |

It showed that lnILI series of Luzhou, Neijiang and Zigong were all stable, then we could fit the VARMA model.

**Table A82 the two-way *p* value table for extended cross-correlation matrix of Luzhou, Neijiang and Zigong**

| **AR order: *p*** | **MA Order: *q*** | | | |
| --- | --- | --- | --- | --- |
|  | **0** | **1** | **2** | **3** |
| **0** | 0.0000 | 0.0000 | 0.0000 | 0.0000 |
| **1** | 0.0000 | 0.1317 | 0.3060 | 0.3242 |
| **2** | 0.2085 | 0.9629 | 0.3014 | 0.7928 |
| **3** | 0.7430 | 0.7532 | 0.8930 | 0.9095 |

As can be seen from the table, the best order was *p*=2 and *q*=0, so we chose VARMA (2,0) for model fitting.

**Table A83 VARMA (2,0) first-order AR matrix of Zhangzhou, Neijiang and Zigong**

| **Dependent variables** | **Independent variables** | | |
| --- | --- | --- | --- |
|  | **Luzhou** | **Neijiang** | **Zigong** |
| **Luzhou** | 0.85 | — | — |
| **Neijiang** | — | 0.94 | -0.02 |
| **Zigong** | — | — | 0.89 |

**Table A84 VARMA (2,0) second-order AR matrix of Zhangzhou, Neijiang and Zigong**

| **Dependent variables** | **Independent variables** | | |
| --- | --- | --- | --- |
|  | **Luzhou** | **Neijiang** | **Zigong** |
| **Luzhou** | 0.15 | — | — |
| **Neijiang** | — | 0.74 | -0.31 |
| **Zigong** | — | — | 0.22 |

**21) Luzhou, Yibin, Zigong parameters learning results:**

**Table A85 the unit-root test result of Luzhou, Yibin and Zigong**

| **lnILI** | **Dickey-Fuller** | ***P* value** |
| --- | --- | --- |
| Luzhou | -4.9132 | ＜0.01 |
| Yibin | -3.4497 | 0.04767 |
| Zigong | -4.8704 | ＜0.01 |

It showed that lnILI series of Luzhou, Yibin and Zigong were all stable, then we could fit the VARMA model.

**Table A86 the two-way *p* value table for extended cross-correlation matrix of Luzhou, Yibin and Zigong**

| **AR order: *p*** | **MA Order: *q*** | | | |
| --- | --- | --- | --- | --- |
|  | **0** | **1** | **2** | **3** |
| **0** | 0.0000 | 0.0000 | 0.0000 | 0.0000 |
| **1** | 0.0000 | 0.8606 | 0.8466 | 0.6779 |
| **2** | 0.5775 | 0.9842 | 0.8452 | 0.9944 |
| **3** | 0.8765 | 0.9998 | 1.0000 | 1.0000 |

As can be seen from the table, the best order was *p*=2 and *q*=0, so we chose VARMA (2,0) for model fitting.

**Table A87 VARMA (2,0) first-order AR matrix of Zhangzhou, Yibin and Zigong**

| **Dependent variables** | **Independent variables** | | |
| --- | --- | --- | --- |
|  | **Luzhou** | **Yibin** | **Zigong** |
| **Luzhou** | 0.80 | -0.07 | — |
| **Yibin** | — | 0.88 | — |
| **Zigong** | — | — | 0.90 |

**Table A88 VARMA (2,0) second-order AR matrix of Zhangzhou, Yibin and Zigong**

| **Dependent variables** | **Independent variables** | | |
| --- | --- | --- | --- |
|  | **Luzhou** | **Yibin** | **Zigong** |
| **Luzhou** | 0.14 | -0.04 | — |
| **Yibin** | — | 0.72 | — |
| **Zigong** | — | — | 0.23 |

**22) Meishan, Neijiang, Ziyang parameters learning results:**

**Table A89 the unit-root test result of Meishan, Neijiang and Ziyang**

| **lnILI** | **Dickey-Fuller** | ***P* value** |
| --- | --- | --- |
| **Meishan** | -4.4043 | ＜0.01 |
| **Neijiang** | -4.1211 | ＜0.01 |
| **Ziyang** | -2.005 | 0.5748 |

The lnILI series in Ziyang was not stable, so cointegration test was conducted for detecting cointegration relationship between those series. The result showed that cointegration relationship existed between them, and VARMA model could be fitted.

**Table A89 the two-way *p* value table for extended cross-correlation matrix of Meishan, Neijiang and Ziyang**

| **AR order: *p*** | **MA Order: *q*** | | | |
| --- | --- | --- | --- | --- |
|  | **0** | **1** | **2** | **3** |
| **0** | 0.0000 | 0.0000 | 0.0000 | 0.0000 |
| **1** | 0.0000 | 0.6053 | 0.4954 | 0.5835 |
| **2** | 0.0589 | 0.7783 | 0.3676 | 0.8310 |
| **3** | 0.2825 | 0.9977 | 0.9683 | 0.9094 |

As can be seen from the table, the best order was *p*=1 and *q*=1, so we chose VARMA (1,1) for model fitting:

**Table A90 VARMA (1,1) first-order AR matrix of Meishan, Neijiang and Ziyang**

| **Dependent variables** | **Independent variables** | | |
| --- | --- | --- | --- |
|  | **Meishan** | **Neijiang** | **Ziyang** |
| **Meishan** | 0.92 | — | 0.00 |
| **Neijiang** | — | 0.80 | — |
| **Ziyang** | — | — | 0.90 |

**Table A91 VARMA (1,1) first-order MA matrix of Meishan, Neijiang and Ziyang**

| **Dependent variables** | **Independent variables** | | |
| --- | --- | --- | --- |
|  | **Meishan** | **Neijiang** | **Ziyang** |
| **Meishan** | 0.73 | — | -0.27 |
| **Neijiang** | — | 0.46 | — |
| **Ziyang** | — | — | 0.32 |

**23) Mianyang, Nanchong and Suining parameters learning results:**

**Table A92 the unit-root test result of Mianyang, Nanchong and Suining**

| **lnILI** | **Dickey-Fuller** | ***P* value** |
| --- | --- | --- |
| **Mianyang** | -4.3665 | ＜0.01 |
| **Nanchong** | -3.4922 | 0.044 |
| **Suining** | -4.2645 | ＜0.01 |

It showed that the lnILI series in Mianyang, Nanchong and Suining were all stable, then we could fit the VARMA model.

**Table A93 the two-way *p* value table for extended cross-correlation matrix of Mianyang, Nanchong and Suining**

| **AR order: *p*** | **MA Order: *q*** | | | |
| --- | --- | --- | --- | --- |
|  | **0** | **1** | **2** | **3** |
| **0** | 0.0000 | 0.0000 | 0.0000 | 0.0000 |
| **1** | 0.0012 | 0.1469 | 0.1989 | 0.2493 |
| **2** | 0.6092 | 0.7457 | 0.9844 | 0.9834 |
| **3** | 0.9987 | 0.9973 | 0.9997 | 0.9986 |

As can be seen from the table, the best order was *p*=1 and *q*=1, so we chose VARMA (1,1) for model fitting.

**Table A94 VARMA (1,1) first-order AR matrix of Mianyang, Nancheng and Suining**

| **Dependent variables** | **Independent variables** | | |
| --- | --- | --- | --- |
|  | **Mianyang** | **Nanchong** | **Suining** |
| **Mianyang** | 0.93 | 0.15 | — |
| **Nanchong** | 0.07 | 0.82 | — |
| **Suining** | — | -0.10 | 0.84 |

**Table A95 VARMA (1,1) first-order MA matrix of Mianyang, Nanchong and Suining**

| **Dependent variables** | **Independent variables** | | |
| --- | --- | --- | --- |
|  | **Mianyang** | **Nanchong** | **Suining** |
| **Mianyang** | 0.22 | 0.00 | — |
| **Nanchong** | -0.03 | 0.35 | — |
| **Suining** | — | -0.27 | 0.32 |
